# Supplementary figures and images for: Cold-Sensing TRP Channels and Temperature Preference Modulate Ovarian Development in the Model Organism Drosophila melanogaster
Source: Int J Mol Sci. 2025 Jun 12;26(12):5638. doi: 10.3390/ijms26125638 (PMC12192652; doi:10.3390/ijms26125638)

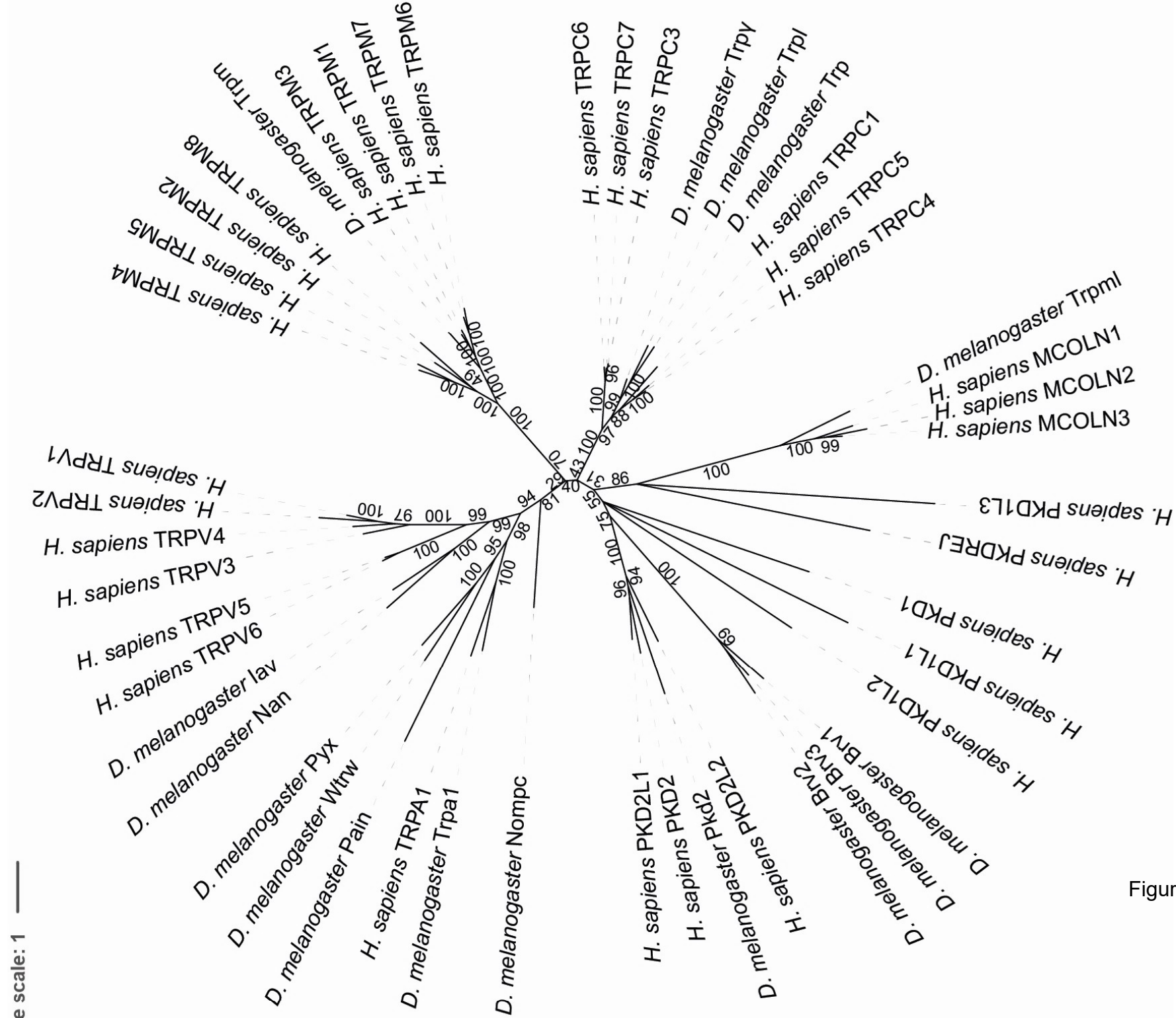

Figure S1

Tree scale: 1

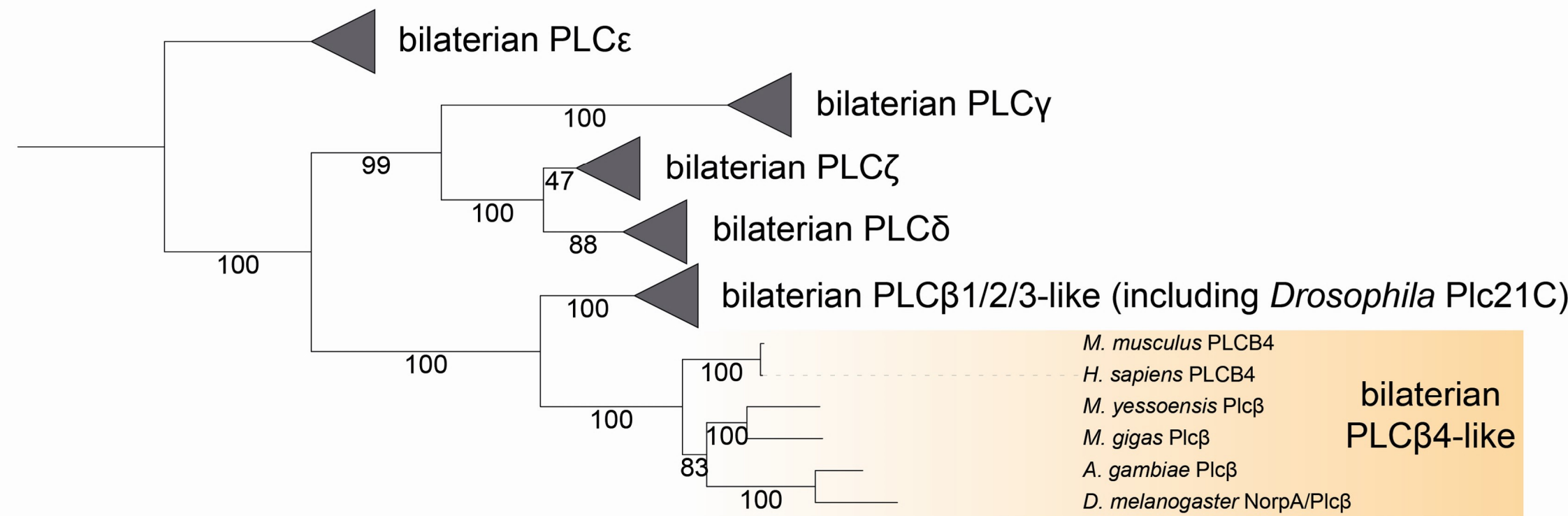

Figure S2

Tree scale: 1

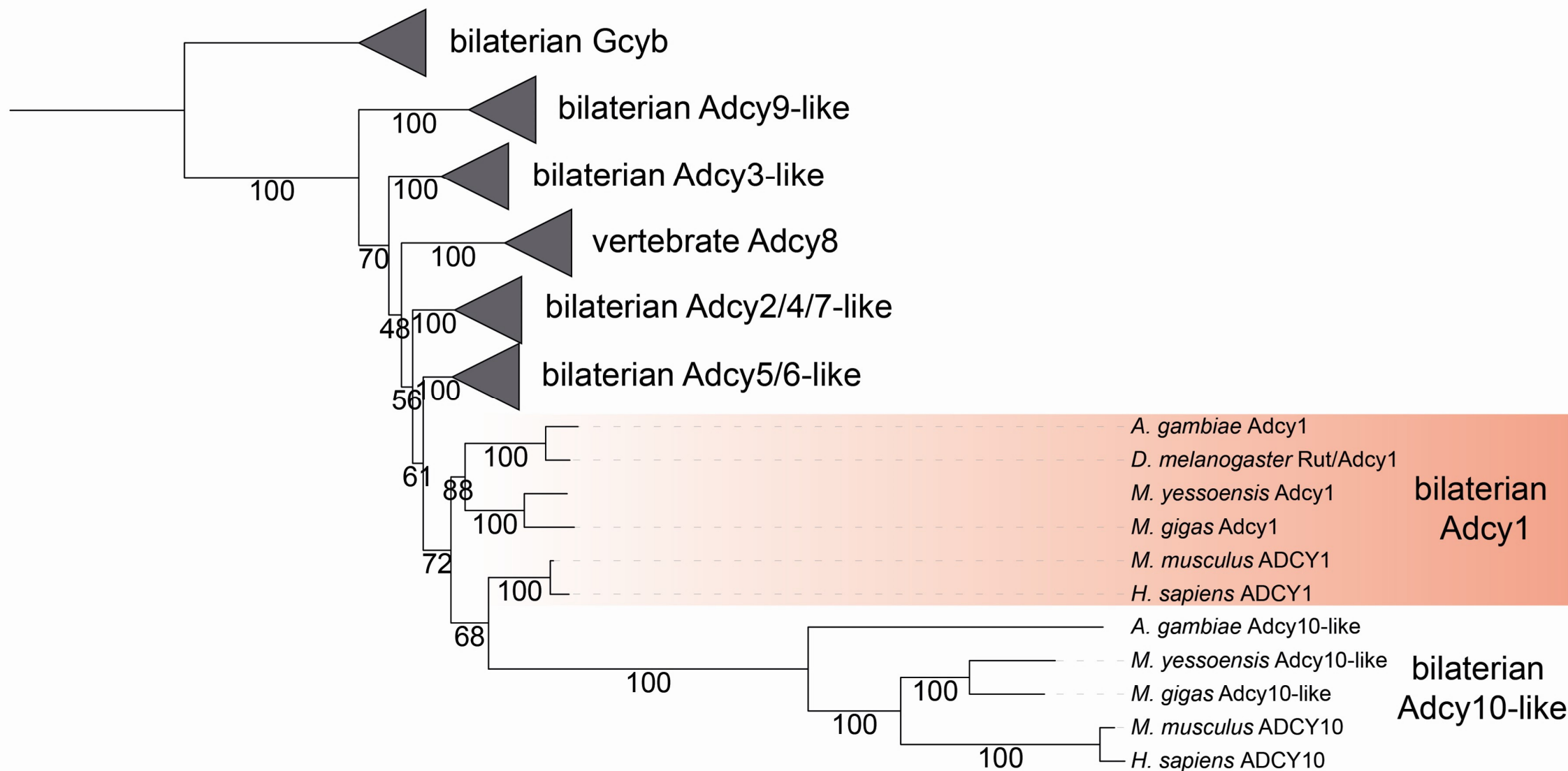

Figure S3

Tree scale: 1

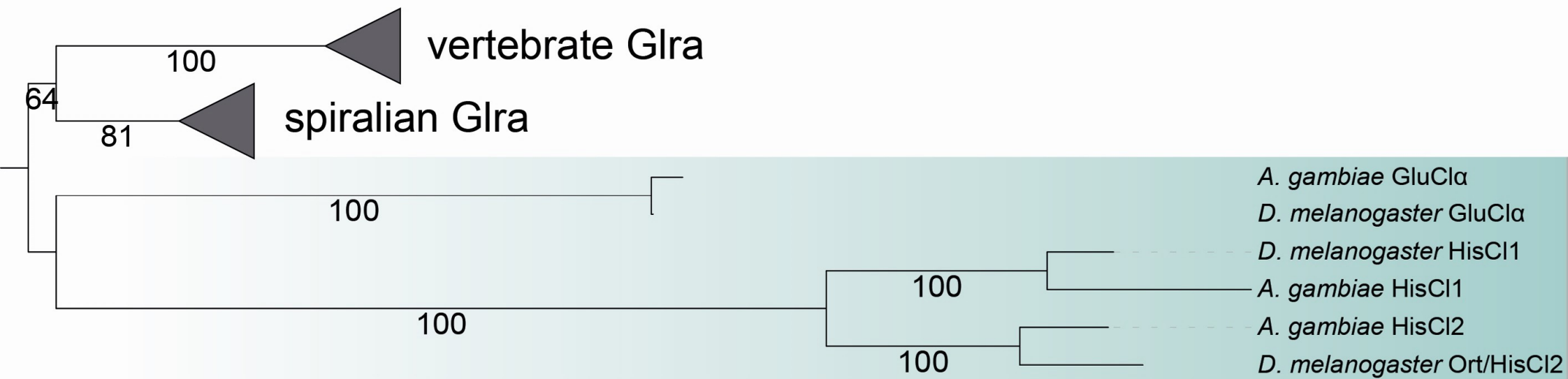

Figure S4

Tree scale: 1

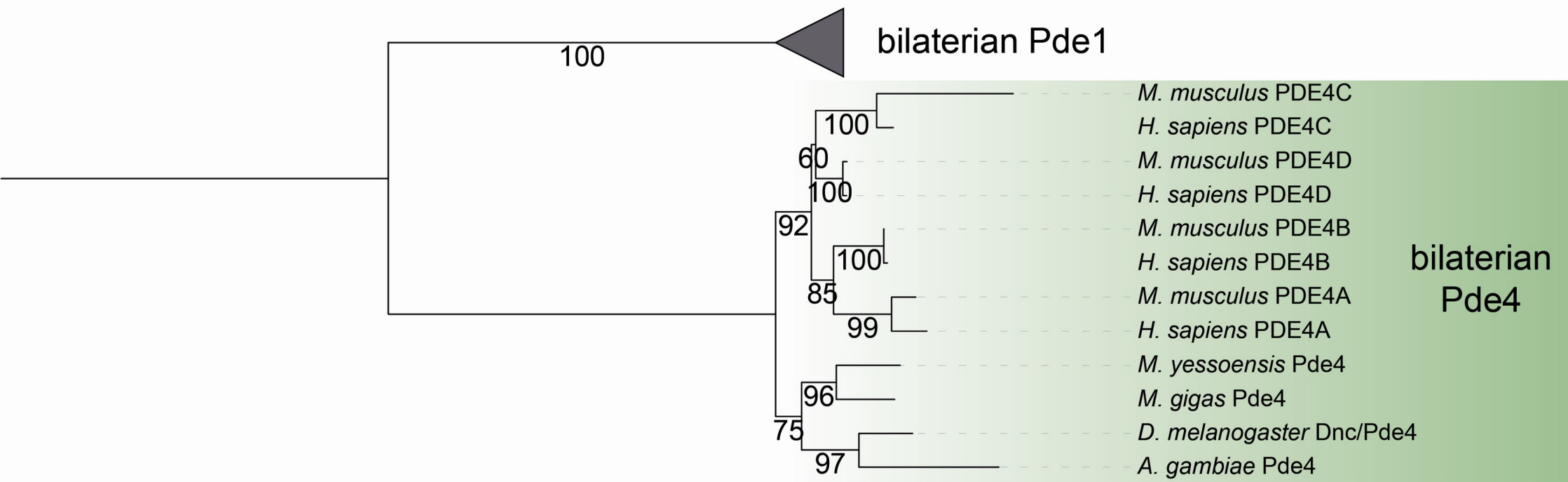

Figure S5

Supplement: Supplementary file 1 [file ijms-26-05638-s001.zip › ijms-3557888-supplementary.pdf]
